# Geographical Inequalities in Use of Improved Drinking Water Supply and Sanitation across Sub-Saharan Africa: Mapping and Spatial Analysis of Cross-sectional Survey Data

**DOI:** 10.1371/journal.pmed.1001626

**Published:** 2014-04-08

**Authors:** Rachel L. Pullan, Matthew C. Freeman, Peter W. Gething, Simon J. Brooker

**Affiliations:** 1 Faculty of Infectious and Tropical Diseases, London School of Hygiene & Tropical Medicine, London, United Kingdom; 2 Department of Environmental Health, Rollins School of Public Health, Emory University, Atlanta, Georgia, United States of America; 3 Spatial Ecology and Epidemiology Group, Department of Zoology, University of Oxford, Oxford, United Kingdom; 4 Kenya Medical Research Institute (KEMRI), Nairobi, Kenya; University of East Anglia, United Kingdom

## Abstract

**Background:**

Understanding geographic inequalities in coverage of drinking-water supply and sanitation (WSS) will help track progress towards universal coverage of water and sanitation by identifying marginalized populations, thus helping to control a large number of infectious diseases. This paper uses household survey data to develop comprehensive maps of WSS coverage at high spatial resolution for sub-Saharan Africa (SSA). Analysis is extended to investigate geographic heterogeneity and relative geographic inequality within countries.

**Methods and Findings:**

Cluster-level data on household reported use of improved drinking-water supply, sanitation, and open defecation were abstracted from 138 national surveys undertaken from 1991–2012 in 41 countries. Spatially explicit logistic regression models were developed and fitted within a Bayesian framework, and used to predict coverage at the second administrative level (admin2, e.g., district) across SSA for 2012. Results reveal substantial geographical inequalities in predicted use of water and sanitation that exceed urban-rural disparities. The average range in coverage seen between admin2 within countries was 55% for improved drinking water, 54% for use of improved sanitation, and 59% for dependence upon open defecation. There was also some evidence that countries with higher levels of inequality relative to coverage in use of an improved drinking-water source also experienced higher levels of inequality in use of improved sanitation (rural populations *r* = 0.47, *p* = 0.002; urban populations *r* = 0.39, *p* = 0.01). Results are limited by the quantity of WSS data available, which varies considerably by country, and by the reliability and utility of available indicators.

**Conclusions:**

This study identifies important geographic inequalities in use of WSS previously hidden within national statistics, confirming the necessity for targeted policies and metrics that reach the most marginalized populations. The presented maps and analysis approach can provide a mechanism for monitoring future reductions in inequality within countries, reflecting priorities of the post-2015 development agenda.

*Please see later in the article for the Editors' Summary*

## Abstract 2

Using cross-sectional survey data, Rachel Pullan and colleagues map geographical inequalities in use of improved drinking water supply and sanitation across sub-Saharan Africa.

*Please see later in the article for the Editors' Summary*

## Editors' Summary

**Background:**

Access to a safe drinking-water supply (a water source that is protected from contamination) and to adequate sanitation facilities (toilets, improved latrines, and other facilities that prevent people coming into contact with human urine and feces) is essential for good health. Unimproved drinking-water sources and sanitation are responsible for 85% of deaths from diarrhea and 1% of the global burden of disease. They also increase the transmission of parasitic worms and other neglected tropical diseases. In 2000, world leaders set a target of reducing the proportion of the global population without access to safe drinking water and basic sanitation to half of the 1990 level by 2015 as part of Millennium Development Goal (MDG) 7 (“Ensure environmental sustainability”; the MDGs are designed to improve the social, economic, and health conditions in the world's poorest countries). Between 1990 and 2010, more than 2 billion people gained access to improved drinking-water sources and 1.8 billion gained access to improved sanitation. In 2011, 89% of the world's population had access to an improved drinking-water supply, 1% above the MDG target, and 64% had access to improved sanitation (the MDG target is 75%).

**Why Was This Study Done?:**

Despite these encouraging figures, the WHO/UNICEF Joint Monitoring Programme for Water Supply and Sanitation (JMP) estimates that, globally, 768 million people relied on unimproved drinking-water sources, 2.5 billion people did not use an improved sanitation facility, and more than 1 billion people (15% of the global population) were defecating in the open in 2011. The JMP estimates for 2011 also reveal national and sub-national inequalities in drinking-water supply and sanitation coverage but a better understanding of geographic inequalities is needed to track progress towards universal coverage of access to improved water and sanitation and to identify the populations that need the most help to achieve this goal. Here, the researchers use cross-sectional household survey data and modern statistical approaches to produce a comprehensive map of the coverage of improved drinking-water supply and improved sanitation at high spatial resolution for sub-Saharan Africa and to investigate geographic inequalities in coverage. Cross-sectional household surveys collect health and other information from households at a single time-point, including data on use of safe water and improved sanitation.

**What Did the Researchers Do and Find?:**

The researchers extracted data on reported household use of an improved drinking-water supply (for example, a piped water supply), improved sanitation facilities (for example, a flushing toilet), and open defecation from 138 national household surveys undertaken between 1991 and 2012 in 41 countries in sub-Saharan Africa. They developed statistical models to fit these data and used the models to estimate coverage at the district (second administrative) level across sub-Saharan Africa for 2012. For ten countries, the estimated coverage of access to improved drinking water at the district level within individual countries ranged from less than 25% to more than 75%. Within-country ranges of a similar magnitude were estimated for coverage of access to improved sanitation (21 countries) and for open defecation (16 countries). Notably, rural households in the districts with the lowest coverage of access to improved water supply and sanitation within a country were 1.5–8 times less likely to access improved drinking water, 2–18 times less likely to access improved sanitation, and 2–80 times more likely to defecate in the open than rural households in districts with the best coverage. Finally, countries with high levels of inequality in improved drinking-water source coverage also experienced high levels of inequality in improved sanitation coverage.

**What Do These Findings Mean?:**

These findings identify important geographic inequalities in the coverage of access to improved water sources and sanitation that were previously hidden within national statistics. The accuracy of these findings depends on the accuracy of the data on water supplies and sanitation provided by household surveys, on the researchers' definitions for improved water supplies and sanitation, and on their statistical methods. Nevertheless, these findings confirm that, to achieve universal coverage of access to improved drinking-water sources and sanitation, strategies that target the areas with the lowest coverage are essential. Moreover, the maps and the analytical approach presented here provide the means for monitoring future reductions in inequalities in the coverage of access to improved water sources and sanitation and thus reflect a major priority of the post-2015 development agenda.

**Additional Information:**

Please access these websites via the online version of this summary at http://dx.doi.org/10.1371/journal.pmed.1001626.

- A *PLOS Medicine* Collection on water and sanitation is available
- The World Health Organization (WHO) provides information on water, sanitation, and health (in several languages)
- The WHO/UNICEF Joint Monitoring Programme for Water Supply and Sanitation is the official United Nations mechanism tasked with monitoring progress toward MDG7, Target 7B; the JMP 2013 update report is available online (also available in French and Spanish through the JMP website)
- The sub-national predictions resulting from this study and the final sub-national maps are available as a resource for researchers and planners

## Introduction

Remarkable gains have been made in the provision of drinking-water supply and sanitation (WSS) globally, with over 2 billion people reportedly gaining access to improved drinking-water sources and 1.8 billion to improved sanitation between 1990 and 2010 [1]. The WHO and UNICEF's Joint Monitoring Programme for Water Supply and Sanitation (JMP) has reported that the world is “on track” to reach the Millennium Development Goal (MDG) target for water supply of reducing by half the proportion of the population without access to sustainable and safe water [1]. In spite of such global gains, important national and sub-national inequalities in WSS coverage remain, including significant rural-urban and regional disparities [1],[2]. National studies also highlight systematic inequalities in WSS access, typically focusing on rural-urban or socio-economic disparities and coverage in hard to reach groups [3]–[9].

Access to safe water and sanitation facilities is a fundamental human right. Unimproved drinking water and sanitation are responsible for an estimated 1% of global disability-adjusted life years (DALYs) [10], and 85% of diarrhoea mortality can be attributed to inadequate water, sanitation, and hygiene practices (WaSH) [11]. Populations with inadequate WSS are also disproportionately affected by the neglected tropical diseases (NTDs) [12]–[17], and as such WSS are increasingly recognised as critical for sustaining the impact of control and elimination strategies that rely on geographically targeted mass drug administration [18],[19]. There have however been few published analyses of sub-national geographical distribution of WSS at policy relevant scales. To better understand inequality within and between countries, and provide a bench mark for tracking progress and help prioritize resource allocation, there is a clear need to develop policy-relevant data on sub-national inequality and a standardised approach to mapping sub-national coverage in WSS.

Population-based national household cluster-sample surveys, such as demographic and health surveys (DHS) and multiple indicator cluster surveys (MICS), provide a wealth of information on the coverage of health and development indicators, including WSS, within countries and have been used previously to map geographical variation in bed net coverage [20], anaemia [21], and under-nutrition [22]. There is an important trade-off however between accuracy and spatial resolution that must be resolved before these data can be truly useful for stakeholders. Surveys are usually powered to provide accurate data for provinces or regions, although access to WSS is likely to vary markedly at this spatial scale. Modern statistical approaches, including small area estimation (SAE) and Bayesian spatial conditional autoregressive (CAR) models, can help tackle the problem of providing feasible estimates for smaller geographical areas, whilst explicitly acknowledging uncertainty associated with data powered to be representative at larger spatial scales [23]–[27].

In this paper we combine multiple national surveys using spatial statistical methods to investigate differences in use of improved drinking water, improved sanitation, and open defecation at small spatial scales; identify specific geographical areas where coverage is substantially worse than national averages; and explore relative geographic inequalities within countries. Our analysis focuses on sub-Saharan Africa (SSA), as this is the region where progression towards the MDGs for water and sanitation is often least successful [1],[28]. Our intent is not to replace existing national JMP estimates, but instead to develop robust maps of contemporary WSS coverage at a finer spatial resolution useful for service delivery providers, policy makers and those planning investment within governments, implementers, and donors. The work is conducted within the context of the Global Atlas of Helminth Infections project (www.thiswormyworld.org), which aims to develop a suite of geographical resources and tools for NTD control.

## Methods

### Data Sources

WSS data were sourced from national household cluster-sample surveys undertaken as part of multiple indicator cluster surveys (MICS) (http://www.childinfo.org/mics4_surveys.html; implementation supported by UNICEF), Demographic and Health Surveys (DHS) and national malaria and AIDS indicator surveys (MIS/AIS) (http://www.measuredhs.com/data/available-datasets.cfm; USAID) and living standard measurement studies (LSMS) (http://iresearch.worldbank.org/lsms/lsmssurveyFinder.htm; World Bank). These surveys are based on probability sampling using existing sampling frames (primarily population censuses) and are conducted by trained enumerators conducting household visits, typically achieving household response rates >97%. Informed consent is obtained from all respondents before participating. All available DHS, MICs, and LSMS surveys conducted in SSA since 1990 that contained the necessary modules to calculate household WSS use were included (*n* = 138 surveys) [29]. Access to improved drinking water and sanitation were defined using the criteria outlined by the JMP, and are measured by reported use. For MDG monitoring, improved sanitation facilities are defined as those that “hygienically separate human excreta from human contact,” whilst improved (“safe”) drinking-water sources are defined as those that are “protected from outside contamination (especially faecal contamination).” The proportion of households reporting open defecation was also recorded. In the subset of data where distance to water source was available (*n* = 121 surveys), we also recorded the proportion of households using an accessible, improved drinking water source. This was defined as one within 1 km (or 15 minutes) of the household, which has been suggested as an appropriate distance for meeting the MDG targets [30]. Further details of indicator definition, and comparison with those used by the JMP, are provided in Box 1.

**Box 1. Comparison with the Joint Monitoring Programme Methodology**

**Data availability**

- Available data sources included in this analysis (DHS, MICs, and LSMS) make up 42% percent of all data in the JMP database for the same countries. Data available to the JMP that could not be used in this analysis include national surveys implemented by governments and census data.

**Common definitions**

- **Improved sources of drinking water:** piped water into the dwelling; piped water to yard/plot/compound; public tap or standpipe; tubewell or borewell; protected dug well; protected spring; rainwater.
- **Improved sanitation:** flush toilet; piper sewer system; septic tank; ventilated improved pit latrine (VIP); pit latrine with slab; composting toilet.
- **Open defecation:** No facilities or bush or field.
- *Not used by JMP.* **Accessible, improved drinking water source:** an improved drinking water source (see above) within either 1 km or 15 minutes from the household. Data available in 85% of surveys.

**Shared sanitation facilities**

- **Contrasting variable definition:** JMP classify all shared facilities as unimproved, and make assumptions on the proportion shared when this data are not available. In our main analysis, we do not distinguish between shared and private sanitation facilities as this information is only available for 70% of included surveys, although a sub-analysis is performed to compare differences
- **Implications:** Coverage of improved sanitation in countries with large numbers of households reporting shared facilities will be considerably higher than current JMP estimates

**Unimproved pit latrines**

- **Contrasting variable definition:** older DHS surveys (phase 4 and earlier, pre-2003) do not distinguish the type of pit latrine (improved or unimproved), and for these surveys JMP make assumptions on the proportion that can be classified as improved. In our analysis all such latrines were included as unimproved to avoid misclassification.
- **Implications:** In our analysis past coverage may have been underestimated for countries with older DHS surveys, and temporal gains in coverage overestimated.

**Methodology**

- **JMP methods:** for each country, separate linear time trends are fit through national (rural and urban) coverage estimates from survey and census data. Regression slopes are extrapolated two years outside available data; beyond this slope is assumed to be zero for a maximum of six years after which estimates are not made. Population averaging (to determine rural, urban, and overall coverage rates) relies on reported survey weights.
- **Spatial multilevel methods:** data from all countries are treated as a continuous time series and fit within a single multilevel model. This considers the hierarchical structure of the whole dataset: surveys sites are nested within administrative areas, within countries. The model estimates an average intercept and an average slope with residual variances across countries, whilst accounting for sub-national spatial correlation and non-spatial variation. In practice, this means that when there is reliable information for a specific country, predictions will closely follow the country survey points, whereas when there is little temporal (or sub-nationally disaggregated) information trends will tend to follow the regional (or national) mean. Population averaging uses high resolution population data generated by the WorldPop project [33],[34].

For each of the available surveys, the proportion of households reporting use of an improved drinking water source, improved sanitation, and open defecation were calculated at the cluster (survey site) level. Each survey site was then coded as either urban or rural as defined by the survey and was allocated, where possible, to sub-national administrative areas. For reference, in most SSA countries first administrative areas (admin1, often referred to as provinces, regions, or states) represent on average approximately 1.35 million people and are typically further subdivided into three to 15 second administrative areas (admin2, often referred to as districts, representing approximately 200,000 people). Admin1 and admin2 digital boundaries were derived for each country using the United Nations Second Administrative Level Boundaries dataset project (SALB) ([31]): for 74 surveys, survey sites were linked to admin2 using the provided survey site co-ordinates, whilst admin1 and admin2 names were used to match survey site locations for the remaining 64 surveys. For 45 of these, survey sites could be matched to admin1 only as admin2 names were not available. In these instances, survey site data were attributed to all admin2 contained within the admin1 boundaries, and weighted to reflect the number of admin2 this represented. No survey data were available for Botswana and Eritrea, and these countries (representing 0.85% of the population of SSA) are therefore not included in the analysis.

Digital gridded population surfaces and urban/peri-urban extent maps for 2012 at 100 m^2^ resolution were provided by the WorldPop project [32],[33]. These population surfaces were used in combination with the admin2 boundaries to extract total admin2 populations and the proportion of each admin2 population living in urban/peri-urban and rural areas. As a confirmation step, the overall proportion urban generated for each country for 2012 was compared with estimates produced by the UN Urbanization Prospects [34] suggesting good agreement (*r* = 0.72, *p*<0.001).

### Data Analysis

For each country, available data for urban and rural populations were plotted on a timescale from 1990 to 2012, and the temporal trend examined visually. Access to improved water and sanitation has risen approximately linearly over time, although there are large differences in this trend between countries, and between urban and rural populations within countries. It was therefore decided that a random coefficient model incorporating a linear time trend would be appropriate, allowing different intercepts and temporal slopes for urban and rural populations by country, but assuming an overall linear change over time.

For each water and sanitation indicator, spatially explicit logistic regression models were therefore developed and fitted using a Bayesian framework. These consider the hierarchical structure of the whole dataset: survey sites are nested within admin2, which are themselves nested within admin1 and countries. Instead of calculating an intercept and slope separately for each country, as is currently done by the JMP, the multilevel model estimates an average intercept and an average slope for rural and urban populations, with residual variances across countries. Neighbouring admin2 within countries are also expected to have similar coverage rates, and so an admin2-level random effect is included that explicitly models the correlation between neighbouring admin2 within countries using a conditional autoregressive (CAR) covariance structure. Models also include a regional random effect with an unstructured covariance structure, to account for non-spatial dependence between survey sites within admin1 areas. A detailed description of the model is given in Text S1.

Essentially, the model structure implies that countries with scarce data over time will follow the mean trend, whilst countries with reliable information (i.e., several surveys across multiple time points) will closely follow the survey points with less associated uncertainty. Similarly, when there is little spatially disaggregated data available, sub-national predictions will follow national means, whilst if data coverage at the admin2 level is good (i.e., survey sites located in admin2 throughout the country) admin2-level coverage will be smoothed towards local, neighbouring values thus counteracting the problem of outliers resulting from under-sampling. Models were fit within a Bayesian framework in the software package WinBUGS [35] using a Markov chain Monte Carlo (MCMC) algorithm, a robust and flexible inference platform that is well suited to complex problems and that explicitly incorporates uncertainty in input data and model parameters. MCMC is an iterative, stochastic simulation technique, and as such this uncertainty is represented in the form of predictive posterior distributions.

### Predicting Coverage in 2012 and Analysis of Geographical Inequality

The developed models were used to predict coverage of water and sanitation for the urban and rural populations of each admin2 for 2012; overall population estimates are population-weighted averages of the urban and rural numbers. Following the approach of Gomez-Rubio and colleagues [24] and Banerjee and colleagues [36], we used information provided by the spatially correlated admin2-level random effect to make predictions for admin2 without data, as detailed in Text S1. To prevent unstable predictions for areas with few or no neighbours with data, this was only performed for countries with available data at admin2 level. For seven countries with no data located at admin2 level, predictions were made for admin1 only. At each MCMC iteration, multiplying the urban and rural population surfaces with the admin2 predicted coverage (or where applicable, coverage by admin1) and then aggregating allowed estimation for the overall population locally and nationally. Resulting uncertainty in predicted coverage will vary geographically, depending on observed sampling variation and the density of survey sites within countries and across time.

Sub-national inequality was evaluated using absolute and relative measures. As absolute measures, the range of coverage (by admin2) for each country was computed, and admin2 with coverage significantly lower (and higher) than the national mean identified based on 95% Bayesian credible intervals (BCIs). As a country-level measure of sub-national relative inequality, we developed a relative geographic inequality (RGI) index, derived from a geographical GINI coefficient. The GINI coefficient is a measure commonly used to describe the distribution of income within a country [37], and has been widely adapted for other applications, including measuring inequalities in health care provision [38],[39] and disease incidence across population groups [40]. It is based on the Lorenz curve, an accumulated frequency curve that compares the distribution of a specific variable with a uniform distribution that represents equality. To calculate Lorenz curves for each country, administrative areas were ranked smallest to largest by their share of national use; the cumulative proportion of use was then calculated and plotted against the cumulative percentage of population. The greater the deviation of the Lorenz curve from the diagonal line of equal distribution, the greater the inequality. The GINI coefficient was then calculated as twice the area between the diagonal and the Lorenz curve. Values can range from 0 to 1, with 0 representing perfect equality and 1 total inequality [37]. Importantly however, in contrast to income, WSS coverage is a bounded variable (i.e., it cannot fall outside the range 0%–100%) and as such the GINI coefficient would be expected to decline as coverage increases. Outlier countries (with higher or lower levels of inequality given their level of coverage) were thus identified using linear regression of GINI coefficient against national coverage for overall, rural, and urban populations and the RGI score generated as the difference between the observed and expected GINI coefficient given national coverage based upon this modelled relationship. This approach draws upon the methods of Jamison and colleagues in their investigation of the relationship between survival curves and life expectancy, which follow similar rules [41]. In the case of open defecation, the GINI and RGI scores were calculated on the basis of households not reporting open defecation (i.e., with access to any type of sanitation) to enable better comparison with the other variables.

### Ethics Statement

This is a secondary analysis of previously collected and published household survey data, and as such ethical approval was not required for this work. Ethical clearance for each included survey was provided by review boards in each country, with informed consent provided by all participants. Details of this process vary by survey.

## Results

### National WSS Coverage

**Figure 1:**
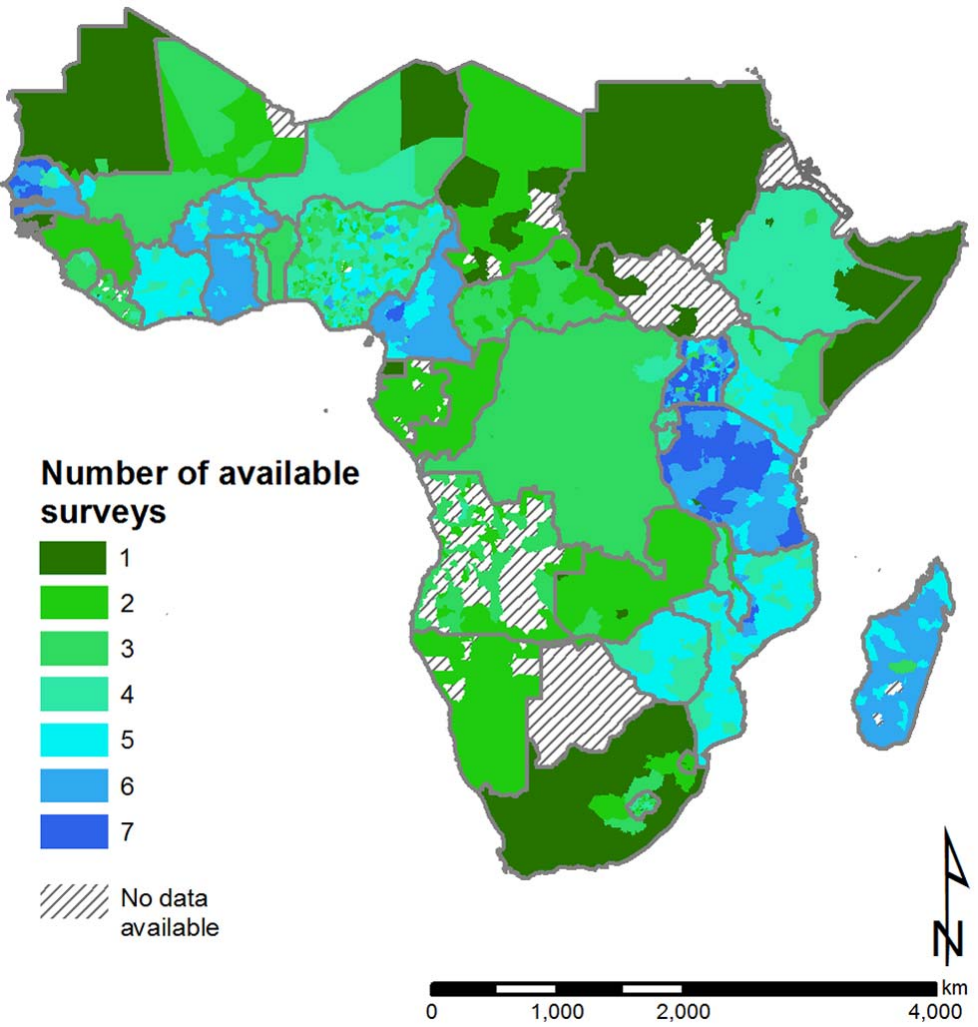
Availability of nationally representative, cluster survey data on improved drinking water and sanitation across sub-Saharan Africa for the period 1990–2012. Data are linked to second administrative areas where possible and if not to the first administrative level; administrative boundaries are provided by the United Nations Second Administrative Level Boundaries (SALB) project.

In total, we obtained 138 DHS, MICs, and LSMS surveys conducted between 1991 and 2012, representing over 1.15 million households from over 50,000 survey sites located in 2,751 admin2 across SSA (outlined in Table 1). Figure 1 emphasises the high data coverage in West and East Africa, and low coverage in much of Southern and Central Africa. Data coverage was very limited for South Sudan and much of Angola.

**Table 1:** Regional summary of water and sanitation coverage data sources and quantity for 41 sub-Saharan African countries.

	Available surveys			Data Located to Second Administrative Area		Data Located to First Administrative Area	
Region	Number by type	Date range		Households	Survey Sites	Households	Survey Sites
Central	11 DHS/MIS/AIS; 6 MICs	1994–2012		38,800	1,434	25,238	993
East	41 DHS/MIS/AIS; 7 MICs; 3 LSMS	1992–2012		294,468	12,573	164,519	8,127
Southern	9 DHS; 1 MICs	1998-2010		61,815	2,796	32,925	1,878
West	43 DHS; 17 MICs	1991–2011		311,136	13,047	254,395	11,036

Sub-Saharan countries not included are Botswana, Cape Verde, Comoros, Djibouti, Eritrea, Reunion, Sao Tome and Principe, and Seychelles, representing <1.0% of the population of SSA in 2012.

AIS, AIDs Indicator Surveys; DHS, Demographic and Health Surveys; LSMS, Living Standard Measurement Studies; MICs, Multiple Indicator Cluster Surveys; MIS, Malaria Indicator Surveys.

**Figure 2:**
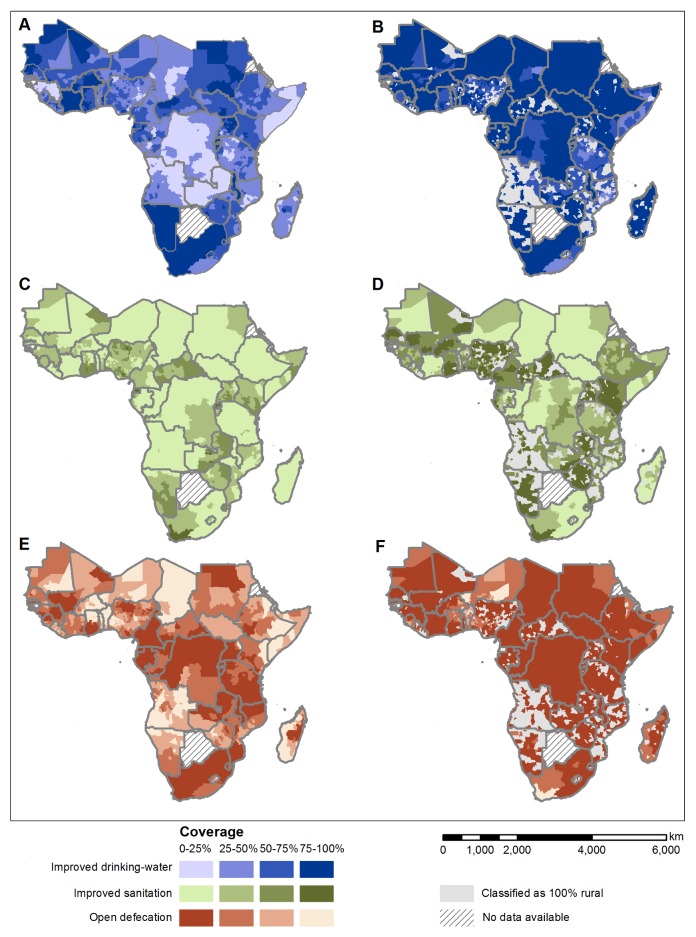
Predicted population coverage in 2012 for rural and urban populations, by second administrative area. Access to improved drinking-water supply in (A) rural and (B) urban populations; access to improved sanitation in (C) rural and (D) urban populations; and open defecation in (E) rural and (F) urban populations. Model results showing posterior median predicted coverage (i.e. most likely value) for each second administrative area. No data was available for Botswana and Eritrea (hatched). Each indicator was modelled independently.

Table 2 provides predicted national estimates and admin2 summaries; the predicted distributions of coverage in urban and rural populations are shown in Figure 2. Model results suggest that in 2010, 62.0% (95% BCI: 61.5%–62.4%) of the population of SSA (excluding Eritrea and Botswana) reported using an improved drinking-water source, although at national levels this was seen to vary between as low as 11.5% (95% BCI: 6.2%–19.0%) in rural Somali populations to 99.0% (95% BCI: 98.7%–99.3%) in urban populations in Namibia. Similar ranges are seen for reported use of improved sanitation: whilst we predict that 42.5% (95% BCI: 42.1%–43.1%) of the total population in SSA used improved sanitation, this ranges from less than 30% for 11 countries to greater than 70% in the top four performing countries (Ghana, Guinea-Bissau, Equatorial Guinea, and Rwanda). For six countries, it is estimated that at least 50% of households habitually defecate in the open (Niger, Namibia, Benin, Burkina Faso, South Sudan, and Chad).

**Table 2:** National estimates and admin2 summaries resulting from the modelling procedures for access to improved drinking-water source, improved sanitation, and open defecation.

Country	Indicator	MDG-7C Achieved^a^ (Yes/No)	National Coverage		Admin2-Level Statistics		RGI Score^c^
			JMP Estimate	Model Estimate (95% BCI)	Range in Predicted Admin2 Coverage	Median Admin2 SD^b^	
**Central SSA**							
Angola	Drinking water	No	53.4%	44.5% (43.4%–45.7%)	9.0%–84.7%	3.2%	**↑** 0.04
	Improved sanitation	No	58.7%	37.5% (36.7%–38.3%)	2.4%–92.8%	2.0%	**↑** 0.18
	Open defecation	—	25.7%	44.3% (43.3%–45.2%)	0.8%–92.8%	3.4%	**↑** 0.08
CAR	Drinking water	No	67.1%	75.1% (73.8%–76.3%)	37.6%–98.6%	2.0%	0.00
	Improved sanitation	No	33.8%	58.3% (56.6%–60.0%)	28.7%–91.8%	2.7%	**↓**−0.06
	Open defecation	—	19.9%	25.2% (23.9%–26.5%)	1.7%–59.1%	1.9%	**↓**−0.02
Congo	Drinking water	Yes	72.4%	74.6% (73.3%–76.0%)	21.2%–87.3%	1.6%	0.01
	Improved sanitation	No	17.8%	16.6% (14.9%–18.5%)	1.4%–22.5%	1.3%	**↓**−0.21
	Open defecation	—	8.3%	10.6% (9.6%–11.8%)	4.4%–40.3%	1.5%	0.00
DR Congo	Drinking water	No	46.2%	43.2% (42.4%–44.1%)	9.0%–93.8%	1.5%	**↑** 0.07
	Improved sanitation	No	30.7%	27.6% (26.7%–28.5%)	7.7%–65.6%	1.7%	−0.01
	Open defecation	—	9.0%	17.3% (16.6%–18.1%)	2.5%–48.2%	1.5%	−0.02
Equatorial Guinea	Drinking water	No		72.8% (39.9%–96.8%)	55.6%–85.1%	18.3%	**↓**−0.03
	Improved sanitation	No		82.0% (34.6%–98.2%)	63.7%–86.6%	16.6%	−0.04
	Open defecation	—		0.2% (0.0%–1.4%)	0.1%–0.2%	0.6%	0.00
Gabon	Drinking water	Yes	87.9%	88.2% (87.8%–88.5%)	30.3%–99.0%	2.1%	**↑**0.05
	Improved sanitation	No	32.9%	53.7% (53.0%–54.5%)	3.9%–79.3%	1.2%	**↑**0.06
	Open defecation	—	1.2%	3.0% (2.7%–3.2%)	0.2%–49.3%	0.4%	0.00
**East SSA**							
Burundi	Drinking water	No	74.4%	78.8% (78.2%–79.4%)	47.9%–95.4%	1.7%	0.00
	Improved sanitation	No	50.1%	40.1% (39.2%–40.9%)	7.9%–88.1%	2.2%	−0.03
	Open defecation	—	2.6%	3.1% (2.8%–3.5%)	0.2%–12.5%	0.5%	0.00
Ethiopia	Drinking water	No	49.0%	62.8% (62.2%–63.4%)	31.5%–99.8%	1.5%	**↓**−0.03
	Improved sanitation	No	20.7%	18.4% (17.9%–19.0%)	4.2%–84.0%	1.1%	**↑** 0.05
	Open defecation	—	45.0%	37.9% (37.2%–38.5%)	3.6%–90.2%	1.6%	**↓**−0.03
Kenya	Drinking water	No	60.9%	71.8% (71.1%–72.6%)	44.7%–95.3%	1.8%	−0.01
	Improved sanitation	No	29.4%	64.5% (63.6%–65.3%)	23.5%–95.4%	2.2%	−0.01
	Open defecation	—	13.7%	16.0% (15.5%–16.7%)	0.1%–83.2%	1.3%	0.02
Madagascar	Drinking water	No	48.1%	44.9% (44.2%–45.5%)	16.4%–96.7%	2.1%	0.01
	Improved sanitation	No	13.7%	7.7% (7.4%–7.9%)	0.7%–44.3%	0.4%	**↑** 0.12
	Open defecation	—	39.4%	43.2% (42.7%–43.8%)	1.3%–88.8%	1.6%	**↑** 0.07
Malawi	Drinking water	Yes	83.7%	82.6% (82.2%–82.9%)	72.8%–96.5%	0.5%	**↓**−0.02
	Improved sanitation	No	52.9%	21.7% (21.1%–22.2%)	9.3%–41.8%	0.8%	**↓**−0.17
	Open defecation	—	6.5%	10.2% (9.9%–10.5%)	3.3%–16.7%	0.5%	**↓**−0.03
Mozambique	Drinking water	No	47.2%	49.5% (48.7%–50.1%)	20.5%–89.7%	2.7%	−0.02
	Improved sanitation	No	19.1%	35.8% (34.9%–36.8%)	5.4%–91.5%	2.9%	0.03
	Open defecation	—	41.8%	24.3% (23.7%–24.9%)	0.5%–55.3%	1.9%	−0.01
Rwanda	Drinking water	No	68.9%	85.6% (85.1%–86.1%)	65.3%–95.8%	1.5%	0.00
	Improved sanitation	No	61.3%	85.0% (84.3%–85.7%)	71.0%–91.3%	1.4%	−0.02
	Open defecation	—	2.2%	1.1% (0.9%–1.3%)	0.1%–4.3%	0.3%	0.00
Somalia	Drinking water	No	29.5%	33.2% (18.5%–45.8%)	3.2%–59.5%	4.4%	0.03
	Improved sanitation	No	23.6%	56.6% (39.5%–78.9%)	6.0%–93.0%	13.5%	**↑** 0.10
	Open defecation	—	52.5%	41.1% (32.9%–47.0%)	7.7%–96.1%	5.2%	**↑** 0.07
South Sudan	Drinking water	No	56.4%	65.5% (24.6%–95.4%)	39.9%–84.7%	23.0%	**↓**−0.08
	Improved sanitation	No	8.9%	27.2% (0.9%–97.6%)	19.0%–28.3%	30.4%	**↓**−0.30
	Open defecation	—	77.0%	61.8% (9.2%–91.7%)	60.6%–68.1%	26.6%	**↓**−0.28
Sudan	Drinking water	No	55.4%	81.0% (55.7%–91.5%)	64.7%–96.7%	13.1%	0.01
	Improved sanitation	No	23.5%	23.2% (1.8%–92.4%)	8.3%–44.9%	32.8%	**↓**−0.13
	Open defecation	—	46.3%	34.3% (17.8%–49.2%)	7.8%–59.5%	9.7%	**↓**−0.04
UR Tanzania	Drinking water	No	74.8%	74.2% (73.8%–74.6%)	18.2%–97.8%	1.8%	**↓**−0.05
	Improved sanitation	No	35.0%	50.9% (50.4%–51.4%)	5.7%–95.1%	2.2%	**↑** 0.06
	Open defecation	—	8.6%	8.6% (8.4%–8.8%)	0.1%–88.0%	0.8%	0.01
Uganda	Drinking water	Yes	53.3%	48.1% (47.5%–48.7%)	18.7%–86.9%	2.2%	**↑** 0.03
	Improved sanitation	No	11.9%	25.7% (25.2%–26.3%)	5.3%–92.4%	1.9%	−0.03
	Open defecation	—	12.4%	13.0% (12.7%–13.4%)	0.1%–50.5%	1.2%	0.01
Zambia	Drinking water	No	64.1%	35.8% (34.6%–37.0%)	4.7%–90.7%	1.5%	**↑**0.15
	Improved sanitation	No	42.1%	62.9% (60.2%–65.4%)	15.6%–92.0%	3.6%	0.04
	Open defecation	—	17.0%	22.8% (21.6%–24.2%)	0.6%–72.6%	2.2%	0.03
**Southern SSA**							
Lesotho	Drinking water	No	77.7%	76.8% (75.8%–77.8%)	44.9%–94.0%	2.9%	−0.01
	Improved sanitation	No	26.3%	37.7% (36.5%–38.9%)	12.6%–72.6%	3.4%	**↓**−0.09
	Open defecation	—	36.8%	37.4% (36.5%–38.6%)	2.2%–88.1%	3.3%	0.01
Namibia	Drinking water	Yes	93.4%	91.6% (90.8%–92.4%)	66.6%–99.7%	1.0%	**↑** 0.04
	Improved sanitation	No	32.3%	47.5% (46.3%–48.8%)	3.7%–94.2%	2.4%	**↑** 0.13
	Open defecation	—	51.6%	51.8% (50.4%–53.1%)	5.5%–94.7%	2.5%	**↑** 0.09
South Africa	Drinking water	No	91.5%	79.4% (49.8%–95.7%)	43.1%–96.4%	11.2%	**↑** 0.03
	Improved sanitation	No	74.0%	45.9% (18.1%–76.3%)	24.3%–92.8%	16.8%	−0.01
	Open defecation	—	7.0%	8.6% (1.9%–23.8%)	1.2%–25.6%	4.9%	−0.01
Swaziland	Drinking water	Yes	72.2%	80.4% (78.3%–82.3%)	55.9%–98.6%	2.5%	0.00
	Improved sanitation	No	57.0%	64.9% (61.4%–68.4%)	43.8%–84.8%	3.7%	**↓**−0.08
	Open defecation	—	14.7%	14.1% (12.7%–15.8%)	0.3%–36.4%	2.3%	−0.01
Zimbabwe	Drinking water	No	80.0%	72.5% (71.8%–73.1%)	48.8%–93.9%	1.7%	0.00
	Improved sanitation	No	40.2%	63.7% (63.0%–64.4%)	21.2%–93.7%	1.6%	0.01
	Open defecation	—	25.6%	27.9% (27.3%–28.5%)	1.2%–77.9%	1.6%	0.01
**West SSA**							
Benin	Drinking water	No	76.0%	72.9% (71.8%–74.0%)	33.0%–99.5%	2.0%	0.01
	Improved sanitation	No	14.2%	51.3% (50.0%–52.8%)	9.4%–95.2%	2.7%	**↑** 0.05
	Open defecation	—	54.3%	55.2% (54.1%–56.4%)	8.8%–94.2%	2.3%	**↑** 0.04
Burkina Faso	Drinking water	Yes	80.0%	84.4% (84.0%–84.8%)	61.4%–94.8%	1.0%	0.01
	Improved sanitation	No	18.0%	39.4% (38.8%–40.0%)	4.7%–80.2%	1.4%	0.01
	Open defecation	—	57.9%	59.1% (58.6%–59.6%)	13.8%–93.6%	1.2%	0.02
Cameroon	Drinking water	No	74.4%	70.5% (70.0%–71.0%)	40.3%–94.8%	1.3%	0.00
	Improved sanitation	No	47.8%	50.7% (50.2%–51.2%)	19.8%–79.2%	1.4%	−0.06
	Open defecation	—	6.2%	6.0% (5.7%–6.2%)	0.4%–26.7%	0.4%	0.01
Chad	Drinking water	No	50.0%	37.3% (33.5%–42.4%)	10.0%–65.4%	2.8%	**↓**−0.09
	Improved sanitation	No	11.7%	1.6% (1.2%–2.2%)	0.2%–8.5%	0.4%	**↑** 0.10
	Open defecation	—	65.4%	82.1% (79.5%–84.1%)	34.6%–97.5%	1.5%	**↑** 0.07
Côte d'Ivoire	Drinking water	No	79.9%	88.3% (87.7%–88.9%)	73.9%–98.0%	1.0%	0.01
	Improved sanitation	No	23.9%	12.4% (11.8%–13.1%)	1.3%–35.4%	0.5%	**↑** 0.06
	Open defecation	—	27.1%	28.6% (27.5%–29.6%)	6.1%–54.5%	2.0%	**↓**−0.03
Gambia	Drinking water	Yes	89.3%	64.8% (52.1%–75.4%)	45.0%–94.6%	2.5%	**↓**−0.03
	Improved sanitation	Yes	67.7%	99.9% (99.8%–99.9%)	99.0%–100.0%	0.0%	0.03
	Open defecation	—	2.3%	1.3% (1.0%–1.7%)	0.0%–8.2%	0.5%	0.00
Ghana	Drinking water	Yes	86.3%	72.0% (71.4%–72.6%)	53.1%–92.4%	1.8%	**↓**−0.03
	Improved sanitation	No	13.5%	71.8% (71.3%–72.3%)	11.6%–94.1%	1.7%	0.03
	Open defecation	—	18.4%	19.2% (18.8%–19.5%)	1.4%–92.3%	1.1%	**↑** 0.04
Guinea	Drinking water	No	73.6%	42.0% (40.1%–44.0%)	12.1%–97.8%	1.9%	**↑** 0.03
	Improved sanitation	No	18.5%	48.0% (44.8%–51.4%)	27.1%–86.2%	3.3%	**↓**−0.06
	Open defecation	—	19.7%	20.5% (19.1%–22.0%)	0.1%–57.5%	1.4%	0.01
Guinea-Bissau	Drinking water	Yes	71.7%	82.6% (42.2%–96.8%)	40.4%–91.3%	20.8%	**↑** 0.03
	Improved sanitation	No	19.0%	80.4% (28.7%–95.1%)	26.9%–90.0%	23.2%	0.03
	Open defecation	—	24.9%	8.3% (2.5%–21.5%)	3.6%–47.9%	12.0%	0.00
Liberia	Drinking water	No	74.4%	67.3% (66.1%–68.6%)	28.4%–88.5%	2.7%	**↓**−0.03
	Improved sanitation	No	18.2%	32.3% (31.2%–33.5%)	5.8%–62.1%	1.9%	−0.03
	Open defecation	—	44.3%	46.3% (45.2%–47.5%)	20.7%–83.9%	2.7%	**↓**−0.05
Mali	Drinking water	No	65.4%	67.9% (67.0%–68.8%)	46.0%–95.4%	1.5%	−0.02
	Improved sanitation	No	21.6%	42.5% (41.4%–43.7%)	13.2%–93.8%	1.8%	0.00
	Open defecation	—	13.5%	16.4% (15.8%–17.1%)	0.1%–61.2%	0.8%	0.01
Mauritania	Drinking water	No	49.6%	59.7% (52.4%–67.9%)	42.0%–87.1%	4.5%	**↓**−0.05
	Improved sanitation	No	26.6%	31.2% (12.4%–44.6%)	2.0%–48.4%	6.8%	0.01
	Open defecation	—	50.9%	39.6% (25.6%–46.5%)	22.9%–85.9%	6.9%	**↓**−0.03
Niger	Drinking water	No	50.3%	57.7% (55.8%–59.5%)	39.2%–90.7%	2.2%	**↓**−0.06
	Improved sanitation	No	9.6%	4.1% (3.7%–4.5%)	0.4%–30.6%	0.3%	**↑** 0.13
	Open defecation	—	78.1%	80.1% (79.1%–81.0%)	0.1–99.8%	1.8%	**↑** 0.06
Nigeria	Drinking water	No	61.1%	59.7% (59.1%–60.2%)	16.5%–91.4%	2.7%	−0.01
	Improved sanitation	No	30.6%	60.7% (60.2%–61.2%)	10.0%–95.9%	2.6%	0.03
	Open defecation	—	22.7%	29.2% (28.7%–29.5%)	0.6%–88.4%	2.4%	**↑** 0.02
Senegal	Drinking water	No	73.4%	78.6% (78.2%–79.1%)	28.1%–98.2%	0.8%	**↑** 0.06
	Improved sanitation	No	51.4%	57.9% (57.4%–58.4%)	17.5%–94.7%	0.9%	**↑** 0.06
	Open defecation	—	16.8%	19.2% (18.8%–19.7%)	0.6%–48.9%	0.9%	0.00
Sierra Leone	Drinking water	No	57.5%	61.5% (60.5%–62.5%)	43.1%–84.5%	1.0%	**↓**−0.03
	Improved sanitation	No	12.9%	48.2% (47.1%–49.4%)	31.9%–72.1%	1.1%	**↓**−0.10
	Open defecation	—	27.1%	28.7% (27.6%–29.7%)	11.4%–59.5%	0.9%	**↓**−0.03
Togo	Drinking water	No	59.0%	58.5% (57.4%–59.7%)	26.1%–79.3%	1.5%	−0.01
	Improved sanitation	No	11.4%	37.4% (36.3%–38.4%)	2.9%–76.7%	1.0%	**↑** 0.10
	Open defecation	—	53.7%	41.2% (40.4%–42.0%)	7.0%–87.5%	1.1%	**↑** 0.03

a MDG 7C is to halve the proportion of the population without sustainable access to safe drinking water and basic sanitation. Classification of achievement is based on figures produced by the WHO/UNICEF JMP for Water Supply and Sanitation for 1990 and 2010 [91].

b Median administrative area standard deviation (SD) is taken from the posterior distribution for standard deviation at the administrative area level generated by the hierarchical model, and reflects the amount of data available for a given country (increased SD reflects greater uncertainty and less data).

c RGI is a measure of relative inequality in access sub-nationally within a given country given national coverage levels. Negative values indicate a lower than expected inequality, whilst positive values indicate greater than expected inequality; **↓** indicates a score significantly lower than 0 and **↑** indicates a score significantly higher than 0.

**Figure 3:**
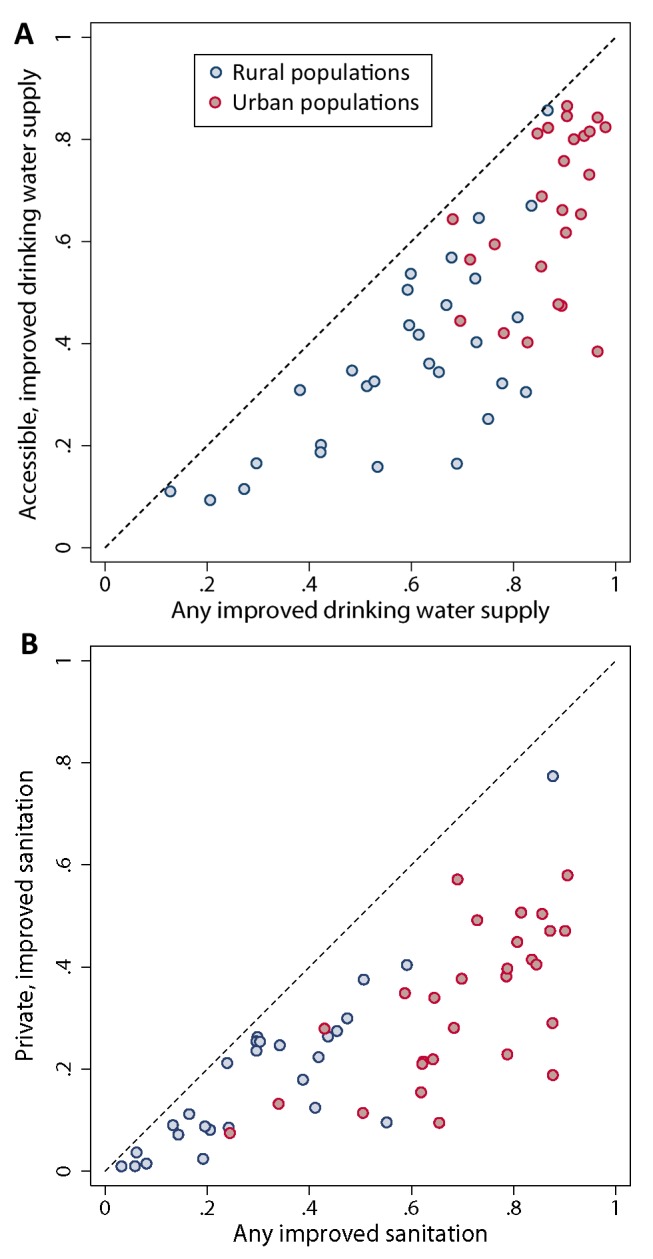
Comparison of (A) any improved drinking water source against accessible, improved drinking water source and (B) any improved sanitation against private improved sanitation. Dots show national comparisons for urban (red) and rural (blue) populations. An accessible drinking water supply is defined as one within 15 minutes of the household; private sanitation is defined as a facility used by only one household.

For 30 countries there was sufficient survey data to investigate reported use of accessible, improved drinking water supply (defined as those <1 km away or within a 30-minute round trip) and for 27 countries sufficient survey data to investigate reported use of private, improved sanitation (defined as one that is not shared with another household). Overall, in 2012 only 55.0% of rural populations using an improved drinking water source used an “accessible” source (range: 23.9% in Uganda to 98.8% in Namibia) rising to 74.1% of urban populations (range: 39.9% in Cote d'Ivoire to 95.8% in Mali). As can be seen in Figure 3, this urban-rural disparity is less apparent for private and shared improved sanitation: overall, 54.1% of rural populations using improved sanitation had access to a private latrine (range: 12.6% in Liberia to 88.7% in Mozambique) compared with 48.9% of urban populations (range: 14.4% in Togo to 82.7% in Mozambique). Given the reduced data availability for these additional indicators, further analysis at a sub-national level is restricted to use of any improved drinking-water source and any improved sanitation facility.

### Sub-national Variation in WSS Coverage

**Figure 4:**
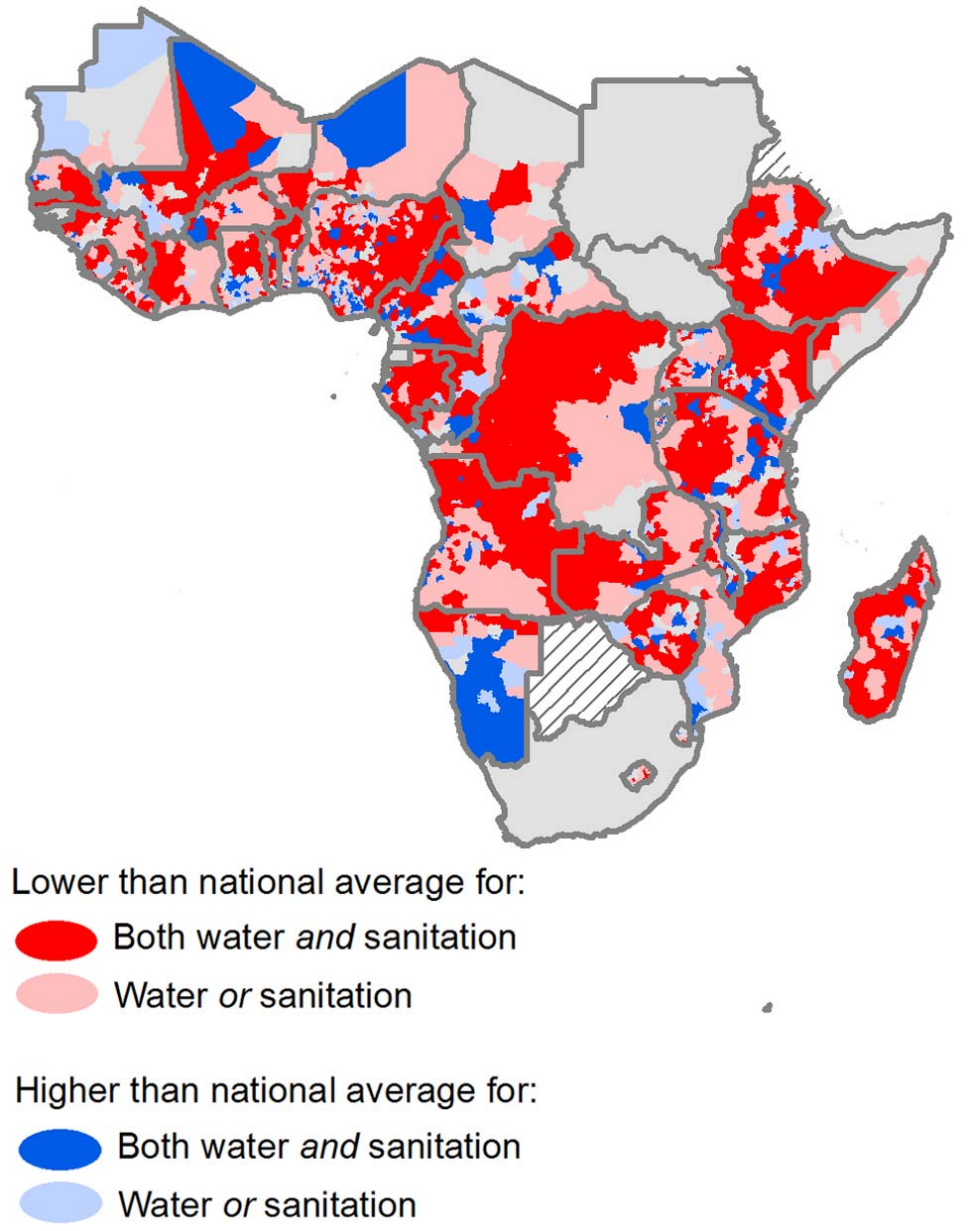
Predicted second administrative areas that differ significantly from national mean coverage. Second administrative areas shaded red have significantly lower coverage than the national average, based on 95% BCI, for either both (dark red) or one (light red) of improved drinking water and improved sanitation; administrative areas shaded blue have significantly higher coverage rates than the national average. Administrative areas shaded grey are not significantly different from the national mean.

**Figure 5:**
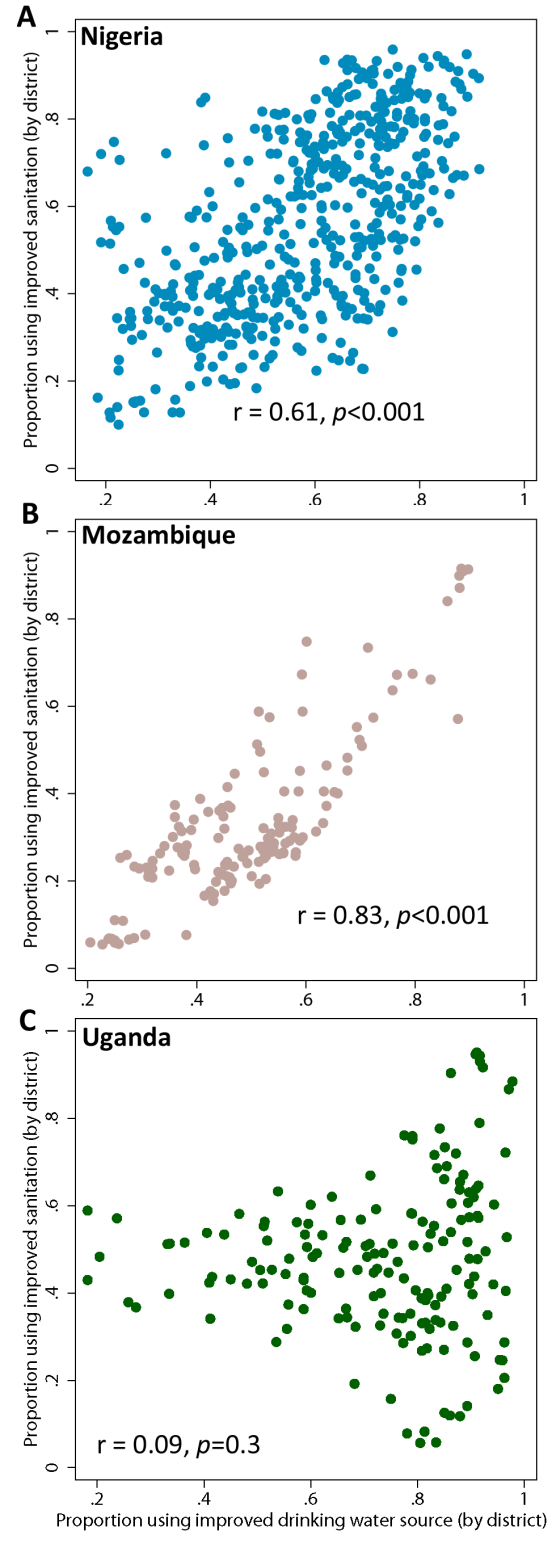
Comparison of coverage in improved drinking water against improved sanitation in overall population, by second administrative area. Comparisons are made for (**A**) Nigeria, (**B**) Mozambique and (**C**) Uganda. *r* is the Pearson pairwise correlation coefficient. Each dot represents one administrative area.

Substantial within-country variation in use is predicted for all three main outcomes, with some of the greatest differences in coverage between admin2 seen for the highly populous countries of Nigeria, Ethiopia, and DR Congo. For ten countries, admin2-level coverage in use of an improved drinking-water source ranged within country from less than 25% to over 75%; within-country ranges of the same magnitude were seen for improved sanitation for 21 countries and open defecation for 16 countries. Figure 4 highlights those admin2 where coverage of both improved drinking water and improved sanitation were significantly lower than national averages based on 95% BCIs (shown in red), highlighting the considerable overlap in admin2 with low access to improved drinking water and improved sanitation. Similarly, when considering all countries in SSA, correlation between admin2-level coverage of improved drinking water and improved sanitation is reasonably high (*r* = 0.44, *p*<0.001) although as can be seen in Figure 5 this varies considerably by country.

**Figure 6:**
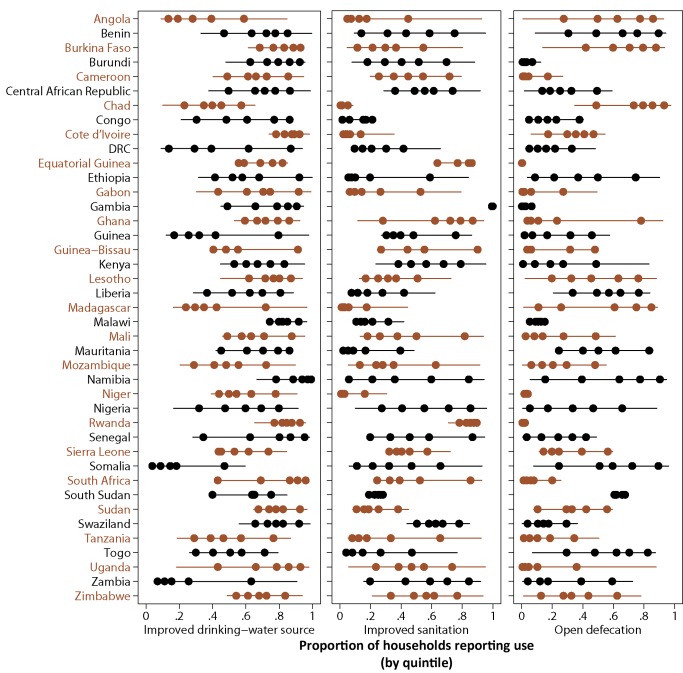
Distribution of modelled WSS coverage across administrative areas by country. Second administrative areas were stratified into quintiles based on coverage of each indicator. Dots show median proportion of households with access for each quintile; lines show the full range in coverage.

The distribution of coverage across all admin2 within each country is shown in Figure 6. For use of an improved drinking-water source, the majority of countries display a linear pattern of coverage, where the distance between quintiles is similar from lowest to highest levels of access. For access to improved sanitation (where coverage is generally lower) a larger number of countries display a top inequality pattern, meaning that coverage in the top quintile is substantially higher than the rest (e.g., Ethiopia, Gabon, Mali, Mozambique, Madagascar, South Africa). In contrast, several countries (including Malawi, Chad, and Sudan) display very little variation between quintiles, especially for access to improved sanitation.

### Inequality Analysis

**Figure 7:**
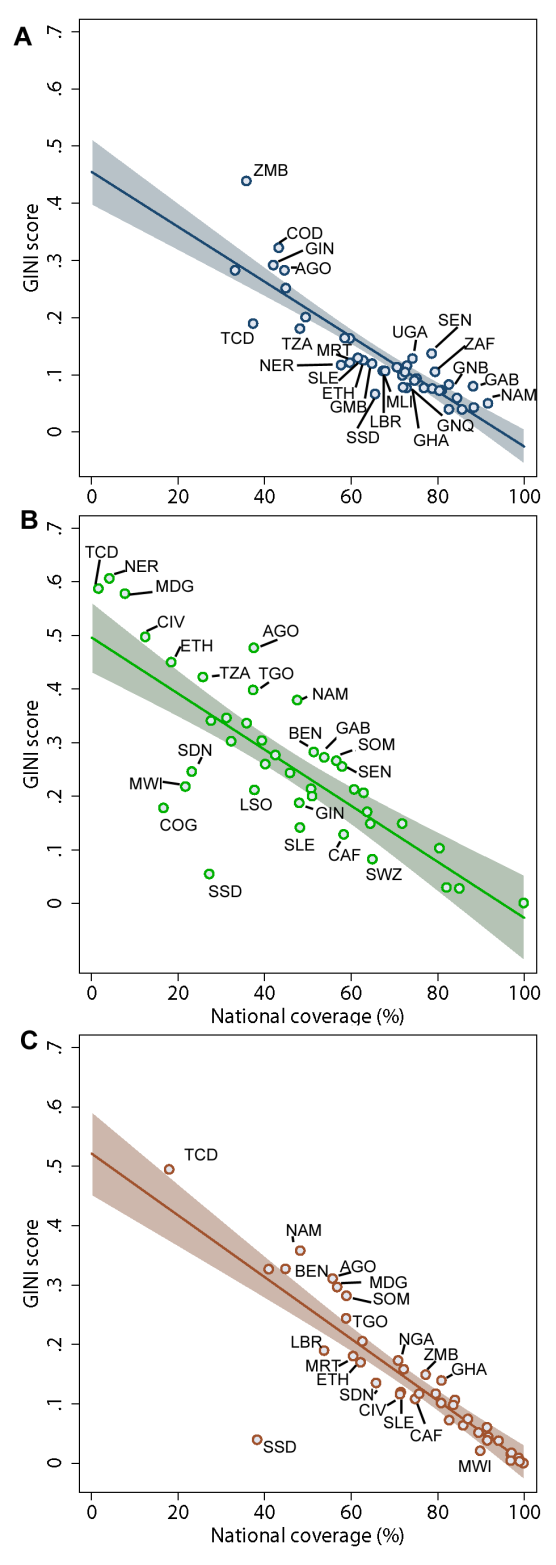
Empirical relationship between inequality (GINI score) as a function of national coverage. Plots are shown for (**A**) use of improved drinking water, (**B**) use of improved sanitation facilities, and (**C**) use of any type of sanitation. All plots show the linear regression prediction (solid line) with 95% confidence interval (shaded area). Labelled countries (by 3-letter ISO codes) are those with GINI scores significantly higher or lower than would be expected, given national coverage.

The degree of geographic inequality in access to WSS varies substantially across SSA. As would be expected, plotting GINI against national coverage reveals strong negative relationships for all three indicators (range in *r* −0.78 to −0.88, *p*<0.001); the linear regression of GINI scores against national coverage thus provides a straightforward mechanism for identifying outlier countries with lower, or higher, levels of inequality than would be expected given their level of coverage. As shown in Figure 7, there are a number of outliers for all three WSS indicators, most noticeably for use of improved sanitation. For use of improved drinking water and any type of sanitation, higher than expected levels of inequality are seen across the full range of national coverage. In contrast, for use of improved sanitation countries with low national coverage are more likely to have higher than expected levels of geographical inequality. There are also some notable outliers with lower than expected geographical inequality given their national coverage rates, especially in access to improved sanitation. The first of these is South Sudan, although this is most likely due to a paucity of data to adequately describe sub-national variation. However Congo, Malawi, and Sierra Leone all display low geographical inequality in access to both improved drinking water and sanitation.

**Figure 8:**
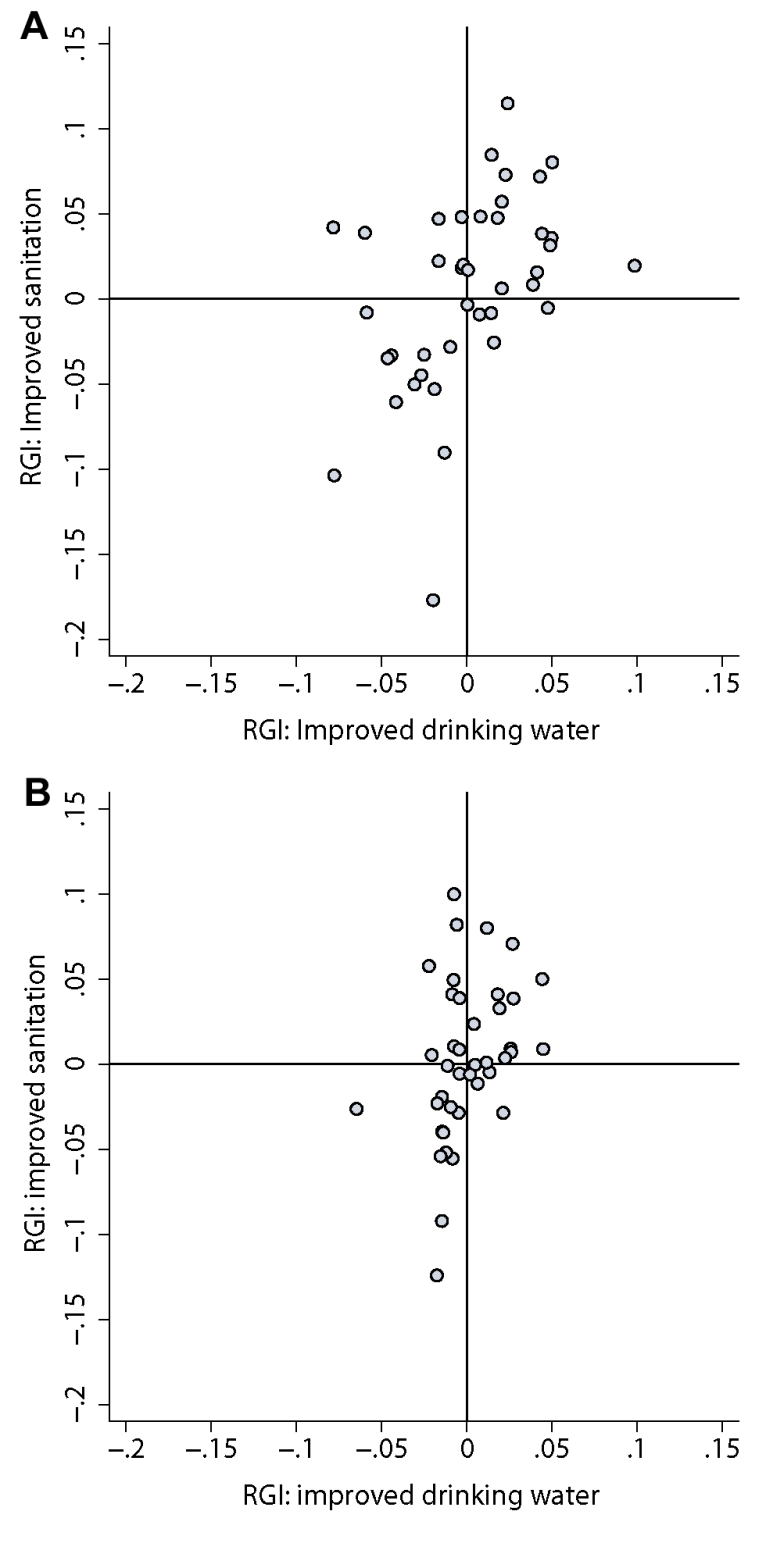
Relationship between relative geographical inequality for use of improved drinking water and RGI for use of improved sanitation for (**A**) rural populations (correlation (*r*) = 0.47, *p* = 0.002) and (**B**) urban populations (*r* = 0.39, *p* = 0.01).

For reference, the difference between the observed and expected GINI scores for the overall population (RGI) are provided in Table 2. There was no evidence for correlation between RGI scores for improved drinking water and sanitation for the overall population at national levels (*r* = 0.18, *p*>0.2). When considering rural and urban populations separately however, there was some evidence that countries with higher levels of relative inequality for use of an improved drinking-water source (i.e., positive RGI scores) also experienced higher levels of inequality for use of improved sanitation (shown in Figure 8; rural populations *r* = 0.47, *p* = 0.002; urban populations *r* = 0.39, *p* = 0.01).

## Discussion

It is already known that coverage of WSS varies considerably between countries in SSA [1]. To our knowledge, this is the first attempt to systematically map and analyse geographical inequalities in WSS within countries. We selected three widely reported WSS coverage indicators (access to improved drinking water, access to improved sanitation, and open defecation) and have generated a new resource for researchers and country stakeholders. Our robust analytical approaches, which explore geographical heterogeneity and geographic inequalities, showed consistent findings for all the three indicators that have important implications not only for the WSS sector, but also the international public health and development communities, policy makers, and donors.

In October 2013, key sector stakeholders issued a joint statement calling on the international community to ensure that the post-2015 development agenda be framed around the principle of equality, emphasising that future goals, targets, and indicators must rely on disaggregated data to allow inequalities to be effectively measured [42]. Our results suggest that there are substantial geographical inequalities in use of WSS across SSA that surpass simple urban-rural disparities and are of similar magnitude to the large socio-economic inequalities highlighted in a number of national studies [2]–[8],[12],[43]–[45]. In almost all countries, rural households in lowest coverage admin2 were 1.5 to 8 times less likely to access improved drinking water, 2 to 18 times less likely to access improved sanitation, and 2 to 80 times more likely to defecate in the open than rural households with the best access. Even across urban populations, coverage in use of improved drinking water varies on average by 30% nationally, use of improved sanitation by 5%, and dependence upon open defecation by 26% nationally, emphasising how population growth and rapid urbanisation may frequently outstrip service provision in poorer urban areas [46]. Considerable overlap in those admin2 with significantly worse coverage than the national average for both improved drinking water and improved sanitation suggests that vulnerable and marginalized populations often suffer the compounded effects of unsafe water and poor sanitation.

Perhaps our most striking finding, however, is that high geographical inequality (i.e., higher than would be expected given absolute national coverage) was seen across all levels of national coverage, although for improved sanitation this was typically greater than would be expected for those countries with lowest national coverage. In addition, those countries with high levels of relative inequality in improved drinking water also experienced higher levels of relative inequality in use of improved sanitation. This finding suggests that countries struggling to increase coverage often also struggle with issues of poor targeting of resources, or patchy implementation of government or NGO delivered interventions, and need to develop strategies and investment plans with reduction of inequalities in mind. Notably, evidence from across the public health and development arena suggests that, unless governments and stakeholders deliberately adopt strategies aimed at reaching lowest coverage areas and population groups, it is unlikely that countries will achieve universal coverage [43],[47]–[49]. It is likely that areas without access to improved water supply and basic sanitation are not only the poorest, but most challenging in terms of environmental conditions and demand for resilient infrastructure, such as low or inconsistent rainfall, poor soils and deep water tables, and a paucity of available markets and materials. The publication in 2012 of the first quantitative aquifer storage maps for the African continent reveals that most countries have the potential for sufficient well-placed hand-pumps to support rural populations; however, the potential for higher yielding boreholes (required to support rapid urbanisation) is limited and will require careful planning [50]. We hope that the information presented here, when combined with quantitative mapping of groundwater resources at sub-national levels, can provide a useful tool to accurately target WSS investments geographically to those admin2 where they are most needed. These data also demonstrate the promise of using geographically disaggregated data as a complementary tool to stratifying on the basis of socio-economic status [51] when systematically measuring progress in reducing inequalities within low coverage countries.

It is commonly recognised that there is no ideal measure for expressing the magnitude of inequalities [37],[52],[53], and as such we have presented both absolute (within country ranges and quintiles) and relative measures. Despite an increasing focus on spatial analysis within health research, there are relatively few publications concerning measurement methods for geographic inequality in health outcomes [54]–[57], and very few papers have explored the use of absolute or relative metrics for sub-national coverage data such as this [58],[59]. We have developed an RGI score, an adaptation of the GINI coefficient. This widely applied coefficient is a ratio analysis measure, and is independent of scale and population size, making it helpful for comparing diverse countries and groups within countries, such as urban and rural populations. It also takes full account of the entire distribution, rather than simply comparing those at the extremes. However, it is a relative measure, and as such countries with vastly differing national coverage can share the same score if the relative distribution is similar. Its use is also limited with bounded variables, such as coverage. Generating an RGI however, which compares the observed GINI coefficient with that expected given existing levels of coverage, helps circumvent the negative correlation that is to be expected when generating GINI coefficients using bounded indicators and allows identification of countries with large relative inequality in provision of WSS. Metrics such as these that accurately align with development principles, including equality of access, are urgently needed. However we would like to add a note of caution: measures of inequality should not be used in isolation, but rather in combination with indicators of absolute coverage. Importantly, equality of use does not reflect how much overall coverage has been achieved, which means that a country could achieve significantly low levels of relative inequality whilst still having very low overall coverage, as clearly demonstrated by Sierra Leone and Ethiopia.

Crucially, our results are entirely dependent upon the quality of the water and sanitation data available. DHS, MICs, and LSMS surveys contain a standardised water and sanitation module in which survey questions and response categories are fully harmonised [29], helping to ensure internal consistency and comparability between countries. However, the only available proxy for sustainable access to improved drinking water source and sanitation is self-reported use and ownership. These reports are rarely if ever supplemented by visual inspection to confirm the functionality and whereabouts of the water source, or correct use of the latrine. Whilst many epidemiological studies rely on self-report to measure type and use of such facilities, to our knowledge, very few studies have examined the reliability of self-reported water and sanitation practices and few have used objective measures to assess actual use [60].

Our analysis is based on those indicators currently recommended for monitoring the MDG targets, but these indicators are subject to considerable debate [61]. For example, not all water sources that are classified as improved will provide water that is safe to drink [62]. Correction with data on drinking water quality would likely have a large impact on estimates, calling into question whether the MDG water target would actually be met by 2015 [61]. For example, the only nationally representative water quality data that exists for SSA, to our knowledge, suggests that only 72% of improved drinking-water sources in Ethiopia were in compliance with WHO and national guidelines on drinking water quality [63]. There is currently massive lack of capacity in many countries to generate reliable and representative information on drinking water quality [64], and so we are unable to make appropriate corrections. Neither have we been able to account for the reliability of water supplies—studies across SSA have suggested that up to around one-third of hand-pumps may be non-functioning—nor of household treatment of water [65]. Our preliminary analysis of distance to water source however has suggested that only a little over half of all rural households reported as using an improved water source were using one within 30-minutes round trip of their household. This is especially striking given that a recent systematic review and meta-analysis has suggested significant increase in illness risk in people living further away from their water source [66]. Distance is an important determinant of the quantity of water brought to the household and used for drinking, cooking, and hygiene behaviours [65], and given our results we would suggest that future monitoring activities should always attempt to quantify the time taken for households to obtain their water.

Similarly controversial is the definition of improved sanitation. For example, a latrine that is correctly used by all members of the household and well maintained can provide an effective barrier to the transmission of faecal-oral disease, whereas a latrine that is not used correctly or that is poorly maintained can actually become a focus for the transmission of disease. Our reported use measures that rely upon the potentially subjective definition of an “improved” pit latrine. Again to avoid approximation, we only include latrines classified as having a slab as improved, which is supported by evidence from Tanzania suggesting the presence of helminth eggs in a large majority (71%) of soil samples collected from more simple pit latrines [67]. Nevertheless, it is not possible to correct for the appropriate use and cleanliness of all latrines classified as improved. Finally, as detailed in Box 1, we have classified all sanitation facilities that use improved technology as improved, regardless of whether they are shared, primarily to avoid approximating when data were unavailable (e.g., all pre-2004 surveys). Although this will include some public sanitation facilities (which may often be used by too many people, be poorly maintained, expensive or distant, and even post a risk for interpersonal violence [68]–[70]), in most instances these represent private facilities shared between five households or fewer. There is little evidence surrounding the health impacts of such facilities [71],[72], but it has been argued that a high proportion are probably safe for health [73]. This will however mean that our estimates of coverage for improved sanitation will be substantially higher than those produced by the JMP, who define all shared facilities as unimproved, preventing them from being directly comparable.

The multilevel modelling approach used here is transparent and flexible, offering advantages over the traditional country-specific linear regression models currently employed by the JMP. As demonstrated in a recent similar exploration of multilevel modelling for estimating national access to drinking water and sanitation [46], using a single model for all countries provides a continuous time series, providing additional information for those countries with scarce data. Our spatial Bayesian approach also enables use of information from neighbouring areas to improve predictions in unsampled areas [24], and quantifies the uncertainty resulting from modelling sub-national estimates at small spatial scales. Some limitations should be noted: we still relied on fitting linear time trends whereas a more flexible curve fitting approach might be more appropriate [46]; we did not include further covariates in the model; we did not consider uncertainties associated with the survey estimates arising from non-sampling errors; and we modelled household, not population, use (i.e., for each survey site we modelled the proportion of households reporting use). The latter may systematically bias our estimates upwards, as it is likely that individuals living in smaller (and potentially richer) households with greater access to WSS will be overrepresented. It will also be important to validate this work as new data become available.

Although not intended to reproduce the JMP, and not directly comparable for improved sanitation, in most instances our estimates are in keeping with official estimates (improved drinking water: *r* = 0.77, root mean squared error [rmse] 11.3%; open defecation: *r* = 0.92, rmse = 7.7%) although they do differ substantially for individual countries, up to 33% for access to an improved drinking-water source and 19% for access to improved sanitation. These differences may be attributed in part to our hierarchical modelling approach—which combines both a more robust handling of temporal trends, and potentially more accurate population weighting as a result of disaggregation. However, the reduced amount of data available for this analysis for many countries (we did not have access to national census data or government surveys) will have played a very important role. For example, the largest difference we see for access to improved drinking water is for Guinea (32%), for which we relied on only two DHS surveys in contrast to the nine surveys and censuses available to JMP.

The implications of geographical inequalities in WSS coverage go beyond logistic considerations of effective service provision. Being able to target where inequalities exist will bring us closer to the goal of universal water and sanitation, which will fulfil human rights obligations. Understanding geographic inequities in WSS coverage can also provide insight into the epidemiology and control of many infectious diseases. Access to safe water and improved sanitation significantly reduce not only diarrhoeal disease [74],[75], but also water-borne diseases such as cholera, typhoid, and cryptosporidiosis [12], and NTDs such as soil-transmitted helminthiasis (STH), schistosomiasis, and trachoma [13]–[16],[76]. In recent years, there has been a concerted effort to develop geographical resources on a range of infectious and tropical diseases [77]–[79] and combining these data with knowledge of local WSS provision and other externalities [18],[80] can provide an empirical basis on which to interpret and predict the impact of public health programmes. For example, there is a large body of evidence that the risk of cholera transmission is greatly reduced as water and sanitation coverage improves [81]–[83], with recent mathematical models suggesting that WSS interventions may be as effective as the oral cholera vaccine in averting cholera cases, with the greatest impacts seen when the two are implemented in combination [84]. Similarly, it is unlikely that drug-based interventions alone will eliminate or control STH, schistosomiasis, and trachoma where WSS coverage remains low [18],[85]–[88]. Whilst growing resolve for collaboration and coordination between the WSS and health sectors is encouraging, there is still a pressing need to build a strong evidence base for collaborative programming [89]. For instance, it has been argued that an historic dissociation of WSS and health sectors has led to major problems in developing and maintaining essential infrastructure [65], and as such the public health community must play a role in setting health-based targets and indicators [51]. An essential component of this is the development of more effective and cross-sectoral coverage and impact indicators [89]. These should be able to effectively measure improvements in both WSS and infection control, and should consider in detail all potential factors that contribute to infection risk.

Although reducing inequalities was not a key element of the original health-related MDGs, there is a growing consensus that monitoring indicators solely at national levels fails to incentivise the targeting of areas of greatest need and potential greatest impact [7],[42],[90]. Here, we have revealed substantial levels of inequality in contemporary access to both improved drinking-water supplies and sanitation and open defecation within countries, and have shown how mapping the geographical distribution of WSS at policy relevant scales can help to make visible those deprived subgroups that were previously hidden within national statistics. As a consequence, we would urge the JMP to consider providing sub-national estimates of coverage within its country profiles, coupled with summary statistics of relative inequality. Mapping a phenomenon, however, does not explain it, and reasons for inequalities are likely to vary substantially between countries. Detailed investigation of the influence of contextual and programmatic factors on contemporary coverage, as well as changing patterns of inequality over time, are outside the scope of the present analysis, but are issues we intend to address in future work at both national and regional scales. More generally, this work has highlighted those countries that are struggling to target resources to areas of greatest need, and it is the responsibility of the international community to provide these countries with assistance in the development of strategies and investment plans to reduce inequality and marginalization. Finally, our intention was to create a vital resource for both researchers and planners, and to this end sub-national maps can be viewed on www.ntdmap.org and the data are available on www.thiswormyworld.org.

## Supporting Information

Text S1 **Technical information: model formulation and predicted coverage.**
(DOCX)

## Abbreviations

BCI: Bayesian credible interval
DHS: demographic and health surveys
JMP: Joint Monitoring Programme
LSMS: living standard measurement studies
MDG: Millennium Development Goal
MICS: multiple indicator cluster surveys
RGI: relative geographic inequality
SSA: sub-Saharan Africa
WSS: water supply and sanitation
