# Supplementary material for: Geographical Inequalities in Use of Improved Drinking Water Supply and Sanitation across Sub-Saharan Africa: Mapping and Spatial Analysis of Cross-sectional Survey Data
Source: PLoS Med. 2014 Apr 8;11(4):e1001626. doi: 10.1371/journal.pmed.1001626 (PMC3979660; doi:10.1371/journal.pmed.1001626)
Supplement: Text S1 — Technical information: model formulation and predicted coverage. (DOCX) [file pmed.1001626.s001.docx]

**Supplementary Information 1: Technical Information**

**Spatially explicit hierarchical model**

For each water and sanitation variable, *s*patially explicit binomial regression models were developed and fitted using a Bayesian framework. These were of the form:

where is the number of households (from a sample of N_ijkl_)reporting access in survey site *i* (conducted in district *j*, province *k*, country *l*), is the intercept of the model, represents correlated random effects on the intercept and temporal slope for each country’s urban and rural populations (such that ), is the year of the survey and is the coefficient representing change in coverage over time, is an unstructured random effect which accounts for province-level variation and is distributed independently as and represents spatially correlated random effects at the district level. This latter random effect was defined using an intrinsic conditional autoregressive (CAR) prior structure, where a simple adjacency matrix was specified with a weight of one given to pairs of districts that shared a common border, and a weight of zero given to pairs of districts that did not share a border, or who shared only a country border.

The model was fitted using Markov chain Monte Carlo (MCMC) in the software package WinBugs [[31](#_ENREF_31)]. Following a burn-in of 9,000 iterations, the values for the intercept and coefficients were stored for 1,000 iterations and model convergence was assessed using diagnostic tests and by visually inspecting the time series plots. Convergence was successfully achieved after 10,000 iterations, and the model was run for a further 10,000 with thinning every ten, during which predictions were made. A sensitivity analysis was performed to determine the most appropriate method for handling missing district references: data points were either: (i) attributed to all districts contained within the province boundaries, and weighted to reflect the number of districts this represented, or (ii) modelled simultaneously without the spatial random effect, and allowed to inform the higher-level components of the model only. Comparison of Deviance Information Criteria (DIC) values, and cross-validation with JMP estimates, suggested that the first approach was the most valid.

**Predicting coverage in 2010 and analysis of geographical inequality**

The developed models were used to predict coverage of water and sanitation indicators for the urban and rural populations of each district for 2012. Following the approach of Gomez-Rubio *et al.* [[21](#_ENREF_21)] and Banerjee *et al.* [[32](#_ENREF_32)], in order to predict coverage for districts without data, we specified a CAR model for the full set of spatial random effects in both districts with and without data, and treated the response data in areas without data as missing, leading to a modified set of full conditional distributions for the spatial random effect. To prevent unstable predictions for areas with few or no neighbours with data, this was only performed for countries with available data at district level. For seven countries with no district-level data, predictions were made for provinces only. At each realisation, multiplying the urban and rural population surfaces with the predicted district coverage (or where applicable, province coverage) and then aggregating enabled estimation for the overall population locally and nationally.
